# The Report on Metropolitan Hospitals

**Published:** 1892-07-02

**Authors:** 


The Hospital. [Extra Special Supplement.

July 2nd, 1892.

### THE REPORT ON METROPOLITAN HOSPITALS

### BY THE LORDS' COMMITTEE.

SUMMARY OF CONTENTS.—Out-Patients—The "Part Pay" System—The Position of the General Practitioner—Remedies Proposed—Suggestions for Reform—Limitation by Inquiry as to Fitness for Admission—Relief of Out-Patient Department through Provident Dispensaries—Use of Out-Patient Department for Consultation—Paying-Patients, and Contributions from the Poor—Unequal Distribution of Hospitals—Proposal to Transplant Hospitals—Want of Co-operation—Hospital Expenditure and Accounts—Food in Hospitals — Nursing — The Committee's Conclusions — Education — Special Hospitals — Poor Law Infirmaries—Hospital Saturday and Sunday Funds—Proposed Central Board—Sources of Maintenance.

After three years' hard work, the Lords' Committee, of which Lord Sandhurst was Chairman, have issued their report on the metropolitan hospitals. It is a very able and full report, exceedingly judicial in tone, and absolutely impartial. Full and grateful public recognition is due to Lord Sandhurst, who has made an excellent Chairman, and devoted a vast amount of time to the subject. The best recognition of the value of Lord Sandhurst's services which the hospital managers could give would be to cordially co-operate with him in carrying out the recommendations of his Committee.

After a few introductory remarks, the report proceeds to deal with the general hospitals, and gives a clear definition of the difference between "endowed" and "voluntary" hospitals, a point on which the public often become confused. There are three endowed hospitals—St. Bartholomew's, St. Thomas's, and Guy's—the first of which derives 93 per cent. of its total income from invested property, and the two latter 75 per cent. In other words, St. Bartholomew's receives 7 per cent., and Guy's and St. Thomas's 25 per cent., of their total income from voluntary subscriptions. There are 16 voluntary hospitals, of which eight have medical schools attached to them. St. George's, the most largely-endowed of voluntary hospitals, derives 71 per cent. of its income from voluntary subscriptions; all the others being almost entirely dependent upon the same source for their revenue. The report next deals with the organisation of individual hospitals, and contains a succinct but exhaustive statement concerning the system of management, resources, and work of each. A statement of the Samaritan funds connected with each hospital is given, from which it appears that, as a rule, although patients are helped in various ways, the chief portion of the revenue is applied for the purpose of sending patients to convalescent institutions.

The admission of patients; in-patients; out-patients; paying patients; the unequal distribution of hospitals; want of co-operation; hospital expenditure and accounts; proposed central board; special hospitals; dispensaries; hospitals and the Metropolitan Asylums Board; Poor-law infirmaries; nursing; medical schools; and the Hospital Sunday and Saturday Funds are all exhaustively treated from various aspects, the evidence given being ably summarised, and its effects impartially weighed and set forth. As these matters occupy upwards of ninety pages of the blue book, it would manifestly be impossible to give the whole of the statement of the Lords' Committee in this special supplement. We are, therefore, compelled, from considerations of space, to publish only the chief and most interesting points, which have a special bearing upon the administration of the general hospitals. We give in the Nursing Mirror a summary of the facts and the findings of the Lords' Committee on the proposed registration of nurses, and have held over, for the present, those portions of the report which deal with special hospitals, dispensaries, Poor-law infirmaries, and medical schools.

It is satisfactory to note that the first edition of the report is already out of print, a fact which shows a widespread interest in the metropolitan hospitals, which, we hope, will bear good fruit. After all, public interest will chiefly centre in the conclusions at which the Lords' Committee have arrived, and the whole of these we have felt it necessary to give.

### Points of Special Interest.

During the course of the inquiry the public have followed with the closest attention (*a*) *the charges made against the London Hospital*, which the Committee, after a somewhat caustic allusion to the authors of these charges, declare, on the whole, to be not substantiated by the evidence; (*b*) *"Truth's" charge against the Treasurer and Governors of St. Bartholomew's Hospital.*—The Committee are of opinion that the system of administration at the endowed hospitals does not, on some points, compare favourably with that at other general hospitals. The neglect to provide a proper system of drainage is, in the opinion of the Committee, the more inexcusable owing to the affluent circumstances of Bart's. They consider the system throws too much power and responsibility into the hands of one individual—the Treasurer. (*c*) *The Proposed Registration of Nurses.*—Although the Committee do not allude to this subject in their conclusions they devote five paragraphs to it, the whole of which will be found reproduced in the *Nursing Mirror*. It will be seen that the Committee are of opinion that the proposed registration is no protection to the public from mere incompetency, and "it was admitted that a woman might go through three years' training at a hospital and get her certificate and yet be a very indifferent nurse, and be known at the hospital to be so; but the public who read her name in the register would suppose her to be competent unless the register clearly stated that it did not guarantee the efficiency of its nurses. On the other hand, if the British Nurses' Association disclaim responsibility for the efficiency of the nurses whom it registers, it seems difficult to understand wherein lies the security which it offers to the public." (*d*) *Pensions for Nurses.*—"The Committee think it very desirable that, where the funds of the hospital permit, pensions should be provided for nurses, whether by the hospital following the example of the London and Guy's, by joining the National Pension Fund for Nurses, or by the hospital providing a special pension out of its own funds."

It thus appears that the Lords' Committee have reported in favour of the position taken up by The Hospital on all the points which it has been our duty to deal with in the interests of the hospitals, the nurses, and the public.

Introductory.

The Select Committee of the House of Lords appointed "to consider the evidence taken during the Sessions of 1890 and 1891 with regard to hospitals and similar institutions within the metropolitan area" have issued their report. The Committee was originally appointed in April, 1890, and the lords named of the committee were the Archbishop of Canterbury, Earl Cadogan, the Earl of Winchilsea and Nottingham, the Earl of Lauderdale, Earl Spencer, Earl Cathcart, the Earl of Kimberley, Lord Zouche of Haryngworth, Lord Clifford of Chudleigh, Lord Sandhurst, the Earl of Erne, Lord Lamington, the Earl of Arran, Lord Monkswell, and Lord Thring. The first blue book was issued in 1890, and the second in 1891. The evidence thus taken is summarised in the pages of the report, and the Committee draw conclusions and make recommendations, which will be found on pages 236 to 240.

### OUT-PATIENTS.

Great Number of Out-patients.

The immense increase in the importance of the out-patient departments of hospitals, and the vast numbers of persons who are now treated in them, give great prominence to this branch of the subject. Taking a few of the large general hospitals, we find that at the London more than 100,000 out-patients are treated in the year (243,000 attendances); at St. Thomas's, 25,000; at the Middlesex, 38,000; at Charing Cross, 21,000; at University College, nearly 40,000; at King's College, 20,000; all these being cases of separate patients, each of whom comes, on the average, three times for treatment, and being, moreover, exclusive of many trivial cases which are not recorded, and also of lying-in cases which are treated outside the hospital. The number of out-patients treated during the year at the 11 hospitals, with schools, was estimated by one witness at over half a million. The most opposite opinions are held as to the usefulness of these departments, and as to the mode in which they are conducted.

Objections to Out-patient Departments.

On the one hand it is urged:—

1. That the number of persons who come for treatment is so great that they cannot be properly attended, and that in consequence,
2. The patients are often wrongly treated, and
3. Are in many cases treated by unqualified students.
4. That the hospitals encourage large numbers to come, in order to raise funds from the public by showing a large total of cases treated.
5. That the hurried treatment has a bad effect on students.
6. That the evils of crowding and hurry are aggravated by the treatment of trivial cases which ought never to come to a hospital.
7. That no sufficient discrimination is used in the admission of out-patients, whereby, and that consequently,
8. Persons are treated free who ought to pay,
9. The poor are pauperised and rendered improvident,
10. Provident dispensaries are stifled,
11. The general practitioner is both deprived of his patients, and
12. Is driven to reduce his fees.

Those, on the other hand, who uphold the efficiency and the usefulness of the out-patient department, maintain that these objections are either exaggerated or totally unfounded; that out-patient departments are of great value to the private practitioner in two ways, by affording him a ready means of obtaining a consultative opinion in a difficult case, and enabling him to send to a hospital a patient who cannot pay his fees; and that, in the interests of medical education, it is absolutely indispensable that such departments should exist.

Some witnesses would abolish the out-patient departments altogether, and these cite the Edinburgh hospitals, which have none; some would reform them, and there are several ways in which they propose to effect the reform; and some are content to let them go on as they are.

Views of Friends, Opponents, and Critics.

The views of opponents and critics, together with the evidence on the other side, may conveniently be considered under the several heads already enumerated.

1. Overcrowding and Hurried Treatment.—That the number of persons who come for treatment to the out-patients' rooms of some of the hospitals from time to time brings a strain on the powers of the staff to deal with them is an undoubted fact. Not only was this stated by witnesses who were avowedly hostile to the system, but it was admitted by several officers on the staff of the hospitals themselves. It seems, however, from the evidence of the latter class of witnesses, that this evil has of late been greatly mitigated by the checks (to be described later) which several of the hospitals have adopted upon the indiscriminate admission of out-patients. It was further asserted by many gentlemen in private practice, some of whom had formerly had hospital experience, that the over-crowding was such that it was impossible to give proper attention to the cases, and it was said that a single doctor would dispose of 60 cases or more in an hour. The charge of hurried treatment seems to have been brought against the hospitals generally, but was especially directed against St. Bartholomew's and the London Hospital. To arrive at any conclusion on the point, it is necessary, first, to see how the out-patient department of a great hospital is worked. The plan adopted is not always the same, but in the larger hospitals the people are generally received, in the first instance, between certain hours in the casualty-room, where they are seen by the assistant or house physician or surgeon (who is assisted in some hospitals by one or more of the advanced students). Many of these cases are of a trivial character, and are disposed of at once. The more serious ones are not treated in this way as "casualties," but are passed on, with a ticket or letter, to the out-patient department proper, where they are seen by the assistant physicians and surgeons. It is thus possible to pass a large number of patients through the casualty-room in a comparatively short time, but the work there is to a great extent merely that of sorting and sending on, while of the slight cases which are at once treated, many require only (it may be) a diarrhœa mixture, or a dressing which is applied under the house surgeon's direction by one of the student "dressers." In some hospitals (*e.g.*, St. Bartholomew's) the patients are first received by members of the junior assistant staff, whose duty is solely to divide them into "casualties" and "out-patients," and to forward them to the proper department for treatment, the casualties being sent to the house physicians and surgeons. This sifting process can, of course, be done very rapidly. At the Charing Cross and Westminster, and some other hospitals, patients are, during half an hour in the day (or other limited period) admitted direct to the out-patient department.

As incidental to the evils of overcrowding, complaints were made that patients were sometimes kept for many hours waiting before they could be attended to, but it is not easy to see how this could be avoided, and it may to some extent have the good effect of keeping away people able to pay their own doctor.

As regards the numbers actually treated by a single doctor in the out-patient department, the evidence from the hospitals themselves does not agree with these allegations of extreme haste in treatment. At Guy's, for instance, we are told that on an exceptionally busy day some 480 cases are treated, but this number includes the casualty cases which are dealt with by the resident staff; for the out-patients proper there are four doctors who are in attendance for about four hours, and of the cases treated by each of them, only about 20 are new cases.

It was denied that at St. Bartholomew's anything like 60 cases were disposed of in an hour by one man. At that hospital, during six days in May, 2,356 medical cases were admitted to the casualty department, or 390 per day; they were attended to by seven doctors, and, deducting the more serious cases, which were drafted off to the out-patient department, it was estimated that three or four minutes were given on the average to each of the remainder. During ten days, the total number of out-patients proper at St. Bartholomew's, was 769 medical (of whom 190 were new), and 449 surgical (of whom 159 were new). From the London Hospital a detailed analysis was given of the work in the out-patient department during a week in May, 1890, from which it appears that new and specially reserved cases were seen on the medical side at the rate of 13 per hour: old cases 33 per hour; on the surgical side, new and reserved cases, 7 per hour; old cases (many of them very trifling) 43 per hour. Sir Andrew Clark, speaking of his own experience at the "London," said that new cases would have 10 minutes or more; but, considering that the vast majority of cases were trivial, and that the patients had only to be told to continue the treatment already prescribed, it was possible, by being methodical, to dispose of a very large number in the course of an afternoon. Evidence denying that the out-patients were treated with undue haste was also received from St. George's, the Middlesex (where 100 new cases come in daily), and other hospitals, and similar testimony was given by a general practitioner.

(2) MISTREATMENT AND (3) TREATMENT BY STUDENTS.—Instances were given by several general practitioners of the alleged wrong treatment of out-patients in hospitals, both through actual mistakes being made, and through trivial cases (*e.g.*, ulcers) being so carelessly attended to that they grew into serious ones. The mischief was mainly attributed to the want of a proper supervision over the students who, it was alleged, are allowed in the crowd and hurry of the out-patient room to treat patients independently of the proper medical staff. At King's College Hospital slight accidents (such as cut fingers) are treated by students; but they are strictly forbidden to take more serious cases without sending for the house surgeon. Much evidence was given of the care and good treatment bestowed on out-patients, and, whatever importance be attached to the particular instances alleged to the contrary, it cannot be held that anything like general neglect was proved against the hospitals under this head.

(4) TENDENCY TO INFLATE OUT-PATIENT DEPARTMENTS AS "BAIT" FOR SUBSCRIPTIONS.—A hospital issuing an appeal to the public naturally lays stress on the amount of work it is doing; and therefore the motive for desiring to treat a large number of out-patients undoubtedly exists. Several witnesses referred in general terms to this tendency as contributing to the existing congestion; but there was little direct evidence on the subject. It was said that the temptation to attract out-patients for the sake of swelling returns is more likely to be felt in the smaller special hospitals than in the great general ones; the latter having so much difficulty in getting through the cases which crowd in for treatment, that the necessity of putting a check on their admission is much more felt than any desire of admitting more.

(5) INJURIOUS EFFECT OF EXCESSIVE NUMBERS ON TRAINING OF STUDENTS.—It is said that "an inordinate number of trivial cases wastes the time of the consultee, wearies the attention of the students, and fosters a habit of hasty diagnosis and careless observation, which tend to erroneous and inefficient treatment." Stated as a general proposition, this quotation from the report of a committee of medical men who inquired in 1870 into the administration of hospitals seems to be unanswerable. Not very much evidence was given on this point, but, in view of the numbers who are treated, it is difficult to believe that under the existing system these tendencies can be altogether avoided. The resident medical officer at the Middlesex Hospital, however, while agreeing that for the purposes of instruction it would be better to limit the number of cases, pointed out that only about a third of the casualties were sent on to the out-patient department, and that on this third alone the students attended for the purpose of receiving instruction from the hospital staff. At St. Bartholomew's also it appears that the students do not attend in the casualty department. At St. Thomas's, where the out patients are limited in numbers, it was considered that an increase in the number would make the instruction worse.

(6) QUANTITY OF TRIVIAL CASES.—The majority of persons who present themselves at the out-patient department of a hospital come with trifling ailments which are quite unsuitable for hospital treatment, uselessly occupying the time and wearying the attention of the medical staff, whose best faculties are needed for cases of serious illness. It was the opinion of more than one witness that a good many people frequented the out-patient room more for the sake of conversation than of medical advice; but it was denied that, so far at least as St. Bartholomew's was concerned, there was much opportunity or inducement for practising this kind of abuse. At Guy's a refreshment bar is established. Mention was made also of a "stock bottle," containing a harmless mixture used for the benefit of that class of patients who are not satisfied to be dismissed without a dose. It was said that a great many applicants needed food and washing, but not medicine.

(7) WANT OF DISCRIMINATION IN ADMISSION OF OUT-PATIENTS.—It is generally agreed that the hospitals are intended for those who are too poor to pay for private medical attendance, but who are not recipients of relief under the poor law; and one witness was of opinion that the working classes themselves have a very clear idea who are fit subjects for hospital treatment. It is, however, charged against the hospitals that no sufficient means are adopted for rejecting those applicants who are not proper objects for charity. This charge is more especially directed against the administration of the out-patient departments. The extent to which the charge is true is a matter of dispute. The various methods adopted or proposed for relieving the congestion in these departments, and preventing their abuse, will be dealt with later; but as long as the out-patient system exists at all it can hardly be expected that any remedy will supply an absolute safeguard against abuse. Meanwhile, in those hospitals which have not adopted any special means for controlling the admission of out-patients, the evidence of the hospital authorities shows that the medical officers are expected to ascertain, by observation and inquiry, as far as they are able, the position of persons applying for treatment, with a view to rejecting those who are unsuitable. Strong opinions were expressed, chiefly by medical men practising in poor districts, who are the persons chiefly interested, that this abuse prevailed to a wide extent. The hospital authorities do not in general deny its existence, but say that it is greatly exaggerated; and believe that only a very small proportion of their patients are in a position to pay doctors' fees; many also are cases requiring the best treatment, such as they could not obtain for the low fees which they are able to pay; and some of the better class of patients are sent by their own doctors for the sake of consultation. The evils said to arise from the abuse (whether it be in fact widely spread or not), together with the evidence bearing on them, are noticed under the five remaining heads, which are in reality only different aspects of the same thing.

(8) PERSONS TREATED FREE WHO OUGHT TO PAY.—Cases were cited of persons in good circumstances applying for and obtaining free treatment. A committee of medical men, some 20 years ago, estimated that a fourth of the out patients could pay for private advice, and half could join provident dispensaries. Instances were given of the admission of domestic servants; of private patients who stated that they had been treated at a hospital; of persons assuming a poorer dress in order to gain admission; of persons in affluent circumstances applying for treatment; of persons so applying in order to save a consultation fee. The assistant surgeon of St. Bartholomew's said that the doctors who are getting 5s. a visit or more very often find that their patients go to the hospitals. But, as stated above, a great deal of evidence was forthcoming to the effect that this kind of abuse was rare.

(9) THE POOR PAUPERISED.—Sir E. Hay Currie was of opinion that "the first thing that makes a man a pauper, so to speak, or makes him realise that he can get something for nothing, is the ease with which he gets medical relief." In this condemnation of free treatment, he included not only the hospitals, but also the free medical order under the poor law, since the latter does not involve any loss of the parliamentary franchise. Other witnesses took a similar view, but the opposite opinion was also held, that the free medical treatment kept a very large number of persons in time of sickness off the parish, and thus saved them from pauperism. It was also said that the system of inquiry adopted at some hospitals, by eliminating unsuitable cases, puts a stop to any pauperising tendency; but this argument is not convincing, because the residuum left after the process of elimination is just the class that is said to be pauperised. At all events, the out-patient departments would seem very largely to relieve the poor law, since the whole number of persons treated under the poor law at dispensaries and at their own homes, does not equal the number of out-patients passing through the London Hospital alone.

(10) Provident Dispensaries Stifled.—It is stated that provident dispensaries do not flourish in the neighbourhood of the general hospitals. The decay of the Marylebone Provident Dispensary, the oldest institution of the kind in London, and formerly a flourishing one, was declared to be simultaneous with the growth of the out-patient departments of the Middlesex and University College Hospitals. Conversely, the opinion was expressed that where the out-patients of a hospital are reduced, there provident institutions are sure to spring up. As an instance of the good effect of such an institution where it has free play, the provident dispensary founded in 1880, at Lewisham, may be cited. The Charity Organisation Society at that place used formerly to give to applicants letters for treatment at the Royal Kent Dispensary, a free institution. Since 1881, in which year fifty-one of these letters were given, the number steadily diminished, till in 1888 there was not one. Evidence on the other side, showing that provident dispensaries can and do in some cases flourish in the neighbourhood of hospitals, was of a less positive character; but one witness thought that a hospital where a strict limit was put on the number of out-patients did not interfere with the provident dispensary at all.

(11) General Practitioners Deprived of their Patients.—A number of medical men in practice in the poorer districts were examined on this point, and were almost unanimous in holding a very strong opinion of the injury caused to their class by what they considered the unfair competition of the hospitals, and this view was held in a modified degree by other witnesses not directly interested. As regards the precise extent of the grievance, or the point at which any competition on the part of the hospitals became unfair, there was less unanimity. Those who held that a person able to pay a small fee to a private doctor had no right under any circumstances to receive treatment, or, at all events, out-patient treatment, in a hospital were met by the objection that such a person might be in need of very special skill or experience, and of such advice as only a hospital or an eminent consulting physician could offer. The suggestion was made that ordinary cases should cease to be treated in out-patient departments, which should be used solely for consultative purposes, or for the treatment of serious cases sent on by private practitioners; the evidence touching this point is mentioned more fully below; but here it may be observed that there was some apprehension in the hospitals lest a feeling of jealousy might, to some extent at least, check the flow from the private practitioners to the hospital of those serious cases which needed special treatment. So far as the medical practitioners are concerned, this limited use of the out-patient department would probably remove their grievance; but the hospitals would still be needed for those who are really too poor to pay private fees, unless, indeed, this class is to be wholly relegated to the provident and poor law dispensaries. And, as already mentioned under (7), the hospitals deny that they treat any but a small minority of patients who could pay private fees.

(12) "Sweating" of General Practitioners' Fees.—The competition of free treatment would naturally tend to drive down the fees of the private practitioner; and this was stated to be an urgent evil, and one which has been going on for 10 or 15 years. But the hostility to the indiscriminate free treatment alleged to be given by the hospitals was less strong than the hostility expressed by some to the provident, and more especially the "part-pay" systems. The line of argument seems to be this: "Of two things, one; either let there be charity, pure and simple, so that the receiver knows what it is before he descends to accept it; or else let people pay for what they get; but do not mix up the principles of charity and of self-support, so that a person believes himself to be supplying his own needs out of his own earnings, when all the time he is really more than half a pauper." This is no argument against the provident system when it is properly carried out and pays its way; but one of the chief objects of attack was the out patient department of the Metropolitan Hospital, in which the provident system has, but it is said only partially, been applied. On the part of the hospital it was admitted that the system did not at present pay, but this, it was said, was, as regards the future, merely a question whether enough subscribers joined; * the number was growing, but must increase much more before the experiment could be pronounced a success. In the meanwhile, no doubt it was kept up out of the charitable resources of the hospital; but it was impossible that a venture of this kind should at once be financially successful; as regards the grievance of the private practitioners it was urged that only the very poor were admitted to the provident department of the hospital, and that the hospital, therefore, was not bringing down to a lower level the class which ought to seek private medical advice, but was operating to raise from pauperism that lower class which would otherwise depend solely on the free treatment offered by charity or the poor law.

The promoters of the scheme fully admitted an obligation to avoid injuring the medical man, and were confident that, with the wage limit which they insisted on, their object was attained. It does not appear that the scale fixed for subscriptions is too low to pay the expenses, provided that there are sufficient subscribers; and, therefore, whatever truth (if any) there may be in the allegation that the system tends to drive down private fees, it is not clear that the objection on principle to "part pay" holds altogether good against the Metropolitan Hospital; though it might be, and in fact was, argued that the application of a part of the general funds of the hospital to make good the deficits of the provident department during its period of probation, is a misuse of those funds, and a fraud on those who subscribe them. The local antagonism to this hospital seems to have been partly, at least, due to the employment of medical men from a distance on the staff, instead of local men.

### THE "PART PAY" SYSTEM.

The system of "part pay" is very common in the special hospitals; Guy's also has adopted it for out-patients, who are invited to contribute something towards the cost of their medicine; but in the general hospitals, which are the chief object of the private practitioners' attack, it is not commonly in use. The upholders of the system urge that it is better for the poor to pay something, if they can afford it, however small, than to pay nothing at all; such payments are good morally for the poor, and good materially for the hospitals, whose financial difficulties might to a great extent be removed by them. It was denied, nor does there seem to be any strong evidence, that in the "part pay" hospitals the free patients were worse treated than the paying ones. In its effect upon private practice, however, it seems impossible to doubt that, unless great care is taken to exclude all but the very poor, this system, so far as it goes, must tend to force down private fees; and the more so if it is true. as alleged, that the poor do not in general appreciate the distinction between paying part and paying the whole, so that, however small the payment is, they imagine themselves to be giving the price of what they receive. According to one view, however, the part-pay system acts as a protection to the local doctor, inasmuch as a patient, if he has to pay in either case, will rather go to his own doctor than go through the discomfort and delay of waiting in the out-patient room of a hospital.

The evils alleged to exist under this head were by some witnesses charged in particular against the special hospitals, where the part-pay system is most prevalent, and where at the same time the greatest want of discrimination is shown in the admission of patients. But a witness from a special hospital thought that the general practitioners favoured his hospital because it did not offer free treatment.

### THE POSITION OF THE GENERAL PRACTITIONER.

To complete the picture drawn by the more extreme opponents of the hospitals, we are told that the general practitioner, impoverished by the loss of his patients and the reduction of his fees, deteriorates in capacity and in character, sets up private dispensaries which he works with the aid of unqualified assistants, and is driven to every

* The Secretary of the hospital, however, did not appear to regard the institution as being in principle self-supporting (Byers, 16,770).

shift for obtaining a scanty livelihood. He suffers, his patients suffer, the poor are pauperised, and the public who subscribe their money to the hospitals are defrauded.

As regards the actual fees charged by general practitioners in the poorer districts, some particulars were given in evidence. Payments are commonly made on a higher or lower scale, according to the circumstances of the patient. Some witnesses mentioned a shilling as their lowest fee, and a guinea for confinements; and thought that people who could not pay that ought to be treated for nothing at a hospital; but it appears that there are doctors who will pay three visits and provide medicine for a shilling. A man with a family, who would pay a shilling for a doctor's fee, would, it was thought, be earning at least 30s. a week.

A witness practising in South London stated that the fees in that district ranged from 2s. upwards. In the East-end it was said that a good living could be made at the rate of a shilling for a bottle of medicine and consultation in the surgery, and 1s. 6d. for a visit to the patient's home and medicine; but it was said that some men would open dispensaries and take sixpenny fees, to the great injury of their brother practitioners, and to the risk of their patients' health.

A witness from the West of London regretted that there was no fixed code of fees; this witness also complained that his practice was injured by the out-patient department, though his lowest usual charge was 3s. 6d. or 5s. a visit, including medicine.

Another witness stated that in a working class and middle class district in North London the fees were 2s. and 2s. 6d. for the working classes; but that of late, in consequence of the increase of hospitals and dispensaries, doctors had been driven to take 1s. and 1s. 6d., some even taking 6d.

A witness, whose practice lay in the neighbourhood of St. Bartholomew's, stated that his average fee was 2s. 6d; the very lowest fee he would take would be 1s. with a bottle of medicine.

### CONCLUSIONS.

That many members of the medical profession are scarcely able to earn a living is not disputed; but how far this fact is due to the action of the hospitals, and how far to other causes, seems less certain. One general practitioner admitted that the existing low scale of fees was due in part to the overcrowded state of the profession. Another did not believe that the free or part-pay hospitals interfered with general practice. At St. Thomas's the experience was that the general practitioners were not anxious to retain surgical cases, but were glad to send them on to the hospitals; and it was thought that the general practitioners in the neighbourhood would be sorry to see the out-patient department closed. Similar evidence was given from other hospitals, and a witness expressed the opinion that the practitioners who were injured by the hospitals were not those whom it was generally desirable to protect.

### REMEDIES PROPOSED.

It remains to consider the remedies proposed for the removal of the abuses and shortcomings alleged against the out-patient departments.

### PROPOSAL TO ABOLISH OUT-PATIENT DEPARTMENT.

Some few witnesses would appear to favour a clean sweep being made of the whole existing system, so as to confine the hospitals solely to the treatment of in-patients. Those, however, who advocated the closing of the out-patient department to general patients, admitted, for the most part, that the hospitals ought to provide for cases of real urgency and for cases recommended by medical men for hospital advice and treatment. These cases would, in their opinion, provide sufficient material for the instruction of the students; and the residue of patients who could not pay for private treatment would be relieved at the provident dispensaries,* or under the poor law. This was the solution proposed, not only by the general practitioners, but also by some advocates of the provident system; while others, again, among both these classes, went a step further in concession, and thought the hospitals should still open their doors to the very poor.

### IMPORTANCE OF OUT-PATIENT DEPARTMENT TO MEDICAL SCHOOL.

The suggestion that it might be expedient to shut up the out-patient departments was rejected with unanimity by all the medical witnesses coming from hospitals having schools attached to them. The out-patient department, they said, was of the utmost importance for the sake of the training it afforded their students. Some eminent hospital physicians were inclined to think that the experience gained in the out-patient room, where the student sees the beginnings of disease, is the most valuable portion of his training, and that the shutting up of this department would be a calamity to the public and disastrous to the art of medicine.

That medical students must have an opportunity, in some way, of studying the phases of disease which are seen in the out-patient rooms was admitted on all sides.* The abolitionists (partial or total) thought that this was merely an affair of organisation, and that the needs of the medical schools would be satisfied either by the cases which would filter through to the hospitals from the private practitioner, or by an arrangement which should give the students access to the provident and poor-law dispensaries, and through them (a point declared to be of much importance) to the sick poor in their own homes. It is evident, however, that the hospitals look with much distrust on the efficacy, from their point of view, of the "filtering" process; and are afraid that the cases which would be the most useful for teaching purposes would not reach them, or would reach them in insufficient number.

The proposal that dispensaries should be brought into co-operation with hospitals by some arrangement of affiliation, and should in this way take the place of the out-patient department, is mentioned elsewhere†; it received some favour as a general theory, but it was objected that hitherto the provident dispensary system had not gained much ground, and was quite inadequate to supply the material necessary for the medical schools. It is difficult, however, to see how the provident system is ever to prosper, unless the hospitals will enable it to do so. It seems that at Edinburgh, where the hospitals have no out-patient department, the students acquire a portion of their training in the dispensaries; but a doubt was expressed whether this would ever be found a convenient arrangement, except in a partial degree, in London.

### SUGGESTIONS FOR REFORM.

Various proposals were made for the reform, as distinct from the abolition (whether with or without a reservation for medically-recommended cases), of the out-patient department, the objects in view being to restrict the admission to those who were proper objects of charity, and to prevent overcrowding. Except in those hospitals which have adopted special measures, the only checks upon an applicant who is not palpably an unsuitable case for free treatment, are the limited time during which the doors of admission are open, and the delay and discomfort which he may have to suffer in the waiting-room before his turn comes for treatment. The means which some of the hospitals have adopted for relieving the pressure, are of three kinds, viz., a special system of enquiry into the circumstances of applicants; a daily limitation on the number of new cases; and the making of a small charge for drugs.

### LIMITATION BY INQUIRY AS TO FITNESS FOR ADMISSION.

This system has been adopted at King's College, St. Bartholomew's, the London, and some other hospitals. At King's College it was instituted in 1876. An officer was specially appointed to take down the names and addresses, and to ask certain questions of the applicants as they came in; then, if he saw occasion, reference was made to the Charity Organisation Society. As a matter of fact, not many cases were so referred; but the mere knowledge that inquiry was made is said to have greatly reduced the numbers. We are told that in 1871 there were 33,111 out-patients; in 1875, 28,232; in 1876, 21,346; in 1880, 14,069. Since then they have again been on the increase, and the

* The question of provident dispensaries is discussed separately.

* Sir M. Mackenzie appeared to attach little importance to the teaching in the out-patient department; but this opinion was opposed to the great mass of the evidence (Mackenzie, 2186, 2293-9).

† See under heading "Dispensaries."

number in 1889 was 18,916, including casualty patients; the latter class, as distinct from out-patients proper, appears to have largely increased in numbers. This system is still in force. Patients, however, are not refused first treatment, but are informed (where it is thought desirable) that inquiry will be made.

At the London Hospital (since 1884) and St. Bartholomew's (since 1883) the system is similar; but at the London it applies only to the out-patients admitted by governors' letters and not to the casualties (at King's College and St. Bartholomew's it applies to both classes). Out of 22,000 cases at the London, it is said that inquiries were made in about 800. At St. Bartholomew's mention was made of 30 persons being challenged in a day; 14,000 were questioned in a year; and 357 were visited at their own homes. Returns were put in of the inquiries made at these hospitals (Appendix G). Sir E. Hay Currie, a strong supporter of the provident system, had no great belief in the efficacy of this system of inquiry. Sir S. Waterlow, on the other hand, speaking of St. Bartholomew's, expressed himself as thoroughly satisfied with the system, and believed that the knowledge of its existence kept many unsuitable people away. But it does not seem to have been proved that the total number of applications had been greatly diminished. One effect of the inquiries is to show how many apparently unfit cases are in reality among those most in need of charitable relief. Evidence as to the working of the system in detail was given by Mr. Nixon, the House Governor of the London Hospital, and his opinion was strongly favourable to its efficacy. At each of these hospitals the work of inquiry is performed by a single officer, who has a salary of about £150. Some other hospitals, without having a special officer for the purpose, seem to enquire more or less systematically into the circumstances of their patients, and recourse is had, in some cases (especially by St. George's) to the Charity Organisation Society. The opinion was expressed that the ordinary staff of the hospital should be quite competent to make the necessary investigations without the aid of a special officer; and that the appointment of such an officer would have little effect, and would be, in fact, a useless expense.

Several general practitioners and others spoke in favour of the special inquiry system, of its good effect at the London Hospital, and of the good use which can be made of the Charity Organisation Society for this purpose; and this society was itself in favour of the general adoption of the system. As a further development of it, the proposal was made that every applicant should be required to bring with him some written recommendation, as a guarantee that he was a proper object of charity. At the Great Ormond Street Hospital for Children, and elsewhere, this plan seems to have been tried, but given up; and mention was made of the great difficulty of effectively working any general system of inquiry.

### Payment from Patients.

This plan, which is in force at Guy's, and has been noticed in connection with the organisation of that hospital, was effective for a time in keeping down the numbers; but they increased again to such an extent that a system of limitation has been adopted in addition to the payment system. At the West End Hospital for Paralysis and Epilepsy it is found that both out-patients and in-patients are not unfrequently willing to make some payment. At St. Peter's Hospital for Stone, the out-patient department is more than self-supporting.

### Limitation of Numbers.

The most effectual check on overcrowding has been found in the plan of taking in no more than a certain limited number of new cases every day. Several hospitals apply this check; but it is not always worked in quite the same way. At Guy's, for instance, it appears to apply both to out-patients proper and also to "casuals," so that, if 60 persons apply for treatment on the medical side, 20 will be sent to the out-patient department, 20 will receive cards to be seen by the house physician, and the remaining 20 will be sent away unless any of them are in need of immediate treatment, in which case the rule is relaxed in their favour.

At St. George's the limit is 15 medical and 15 surgical new cases per day, but other cases, if urgent, are treated by the house physicians and surgeons, irrespective of this limit, which refers to the out-patients proper. The selected cases are examined as to their circumstances by a clerk.

At St. Thomas's there is a similar limit. On the medical side, the number is nominally 20, but with the margin allowed for urgent cases it rises to about 23. The daily average of applicants during 1890 was 51; of the 28 not selected about 14 would be treated as casuals, and given medicine for two days; the remainder would be dismissed. The evidence from the medical staff was that the system worked well, and that no system of special enquiry was needed.

At the Westminster no out-patient officer is obliged to see more than 20 new cases a day; but this rule is not strictly enforced.

At the Royal Free Hospital there is a limit of 25 surgical, and 30 medical, new cases.

Opinions favourable to this system were expressed by medical officers at some other hospitals where it has not been adopted, and also by outside practitioners, and by the Secretary of the Charity Organisation Society.

At the Charing Cross Hospital there is no limit of numbers, and it was said that no difficulty is felt.

### RELIEF OF OUT-PATIENT DEPARTMENT THROUGH PROVIDENT DISPENSARIES.

As already mentioned, one scheme of reform provides for the relief of the out-patient departments by the development of the system of provident dispensaries; but the advocates of that system do not seem to be agreed whether the out-patient departments should be altogether closed (except to recommended cases), or whether their doors should be still open to a class between the provident dispensary and the poor law. It is evident that the latter alternative does not provide an escape from the difficulty of discriminating between different classes and phases of poverty; and would necessitate a very efficient system of inquiry, unless the proposal were adopted of making every applicant bring evidence of his necessity with him.

At the Metropolitan Hospital, where the provident system is in operation, it is said that the number of out-patients is kept within reasonable limits.

One witness considered that it ought to be the duty of the medical staff rigidly to exclude all cases not really needing special hospital treatment, and another would enforce the purging of the out-patient department by means of Government inspection and control.

The Secretary of the Charity Organisation Society advocated both limitation of number, and also investigation of cases, the investigation to be conducted by an almoner who should be an officer of experience in charitable work.

### USE OF OUT-PATIENT DEPARTMENT FOR CONSULTATION.

A good many witnesses, among those who did not propose altogether to close the out-patient department to general patients, were in favour of its being used in an increasing degree for consultation purposes. The utility of the hospital for consultation was, in fact, very generally assented to, as was also the desirability of keeping down the number of trivial cases treated at a hospital; but upon the questions whether a letter from a doctor should be the sole passport for admission, and whether the hospital, having once seen and prescribed for the patient, might go on treating him, or must forthwith send him back to his proper doctor or dispensary, there was less unanimity. Out-patients, it was said, should, as in France and in Scotland, receive advice and prescription, but not as a rule drugs; and it seems that some would have the hospitals receive for treatment (as distinct from advice) only those cases sent for that purpose by a private practitioner or from a dispensary.

Others, while advocating the use of the doctor's letter as a passport to the out-patient room, hold that this principle must not be pressed to the point of excluding the very poor who cannot pay for treatment, or of depriving the hospitals of cases necessary for their schools.

Mention has already been made of the feeling in the hospitals that they would not get a sufficient supply of cases through the private practitioners. The out-patient department is already consultative to a considerable extent, and several witnesses doubted whether it could be made much more so than it is now.

Question of Evening Attendance.

Questions were asked as regards the opening of out-patient departments in the evenings. The advantage to the poor of such an arrangement was recognised; but most witnesses from the hospitals regarded it as hardly practicable to secure the attendance of the medical staff at that time. At the provident out-patient department of the Metropolitan Hospital there is evening attendance; also at the Lock Hospital, where it seems to have largely increased the number of applicants. The managers of the Saturday Fund attach importance to it; and it is one of the objects of the Fund to promote it. A general practitioner expressed himself as much opposed to it on the ground that it would crush out private practice.

Insufficient Accommodation for Out-patients.

The want of sufficient accommopation for out-patients is an inconvenience which under existing circumstances is much felt at some hospitals. At St. George's, which appears to have been among those worst off in this respect, the accommodation is now being enlarged.

### PAYING-PATIENTS, AND CONTRIBUTIONS FROM THE POOR.

The great majority of the general hospitals are absolutely free; no payment being taken either from out-patients or in-patients. As regards out-patients, Guy's, and a few general hospitals without schools, which require a small payment in ordinary cases of 3d. or 6d. to meet the cost of drugs, a requirement, however, which is not insisted on where the patient appears to be too poor, seem to be the only exceptions. The only thing generally asked of out-patients is that they should provide their own bottles for medicine.

The beds are also, as a general rule, quite free, the paying beds at St. Thomas's and Guy's being an innovation introduced to meet the financial difficulties of these hospitals.*

In many hospitals boxes are put up into which patients and their friends can, if they please, drop their contributions. In a few cases, it seems that the habit is to call the attention of the patients to these boxes before they are discharged, and to suggest the propriety of their contributing something to the support of an institution which has befriended them, but in general no such request is made; sometimes patients wish to make a direct contribution to the expenses of their maintenance; but this is always refused. The objections to "part-pay" have been mentioned in connection with the grievances and proposed reforms in the out-patient department. But there appears to be a strong feeling on the other side that the poor who benefit by the hospitals ought to contribute according to their means to their support. This view is held both as a matter of principle (and is indeed the leading principle of the Hospital Saturday Fund), and as a matter of expediency, for it is said that if the hospitals would encourage their patients to help them this source alone would go far to remove their financial difficulties, which at the present time are in some cases great and (it is said) increasing. Help from this source is already forthcoming to a not inconsiderable extent, if the special hospitals and the dispensaries and the convalescent homes be included in the account; the total charitable income of these institutions for 1889 being estimated at £300,000, proprietary income £120,000, and payments by patients £45,000. The share of the general hospitals in the last item would doubtless be very small. The Middlesex Hospital, we are told, derives from £20 to £30 a year from this source; the Royal Free, £20. Sir E. Hay Currie, speaking of the provident system in the out-patient department of the Metropolitan Hospital, the income of which was in 1890 about £800, expressed the opinion that the hospitals could if they chose collect from their patients the balance of money required for their support. Upon the question whether the general adoption of this course would check the flow of subscriptions, one witness at least said he did not think it would.

It must be observed that an impression is said to prevail amongst working men that their individual subscriptions, and the contributions which they make through their provident societies and the Saturday Fund entitle them to use the hospitals as a right.

Paying Wards.

The system of admitting paying-patients at St. Thomas's and Guy's is referred to in connection with the organisation of those hospitals. The principle of payment was supported according to their respective methods, by the supporters of the provident and part-pay systems. A danger to be guarded against is lest paying patients should crowd out, or have a preference over, the poor; and the possibility of its being thought that paying patients or patients paying on a higher scale were better cared for than those who paid nothing or paid less, was mentioned as another objection. The objection of some general practitioners to the system of payment by patients in the wards was similar to their objection to it in the out-patient department. One witness thought that the paying-beds had injured the profession more than the out patient department. When witnesses of this class were questioned as to the case of persons able to pay for their ordinary medical treatment, but unable to meet the cost of a serious and expensive operation, and the special treatment and nursing requisite in such a case, it was generally admitted that a hospital was sometimes the proper place for such persons; but objection was still taken to any direct payment being made for services rendered; the proper course, it was suggested, was for the patient to make a gift in the nature of a thank-offering in return for the charity freely accorded him. Another witness, connected with a children's hospital, was strongly in favour of the establishment of a paying ward, on the ground that the very poor are well provided for, and the rich can take care of themselves, but no sufficient provision is available for the lower middle class, who can pay something, but not the full cost of the best private treatment.

The American System.

Another witness spoke in praise of the American system, the principle of which appears to be that everyone's circumstances should be inquired into, and that he should be called upon to pay according to his means; the system being worked by a committee of visitors, some of whom are constantly on the spot investigating the cases. In the Swedish hospitals it seems that no one is treated free; each patient being charged upon a scale appropriate to his means, and the pauper being paid for by the poor law authorities.

Home Hospitals.

Another suggestion was that there should be a separate class of "home" hospitals for the reception of persons of moderate private means, who are now obliged in some cases to seek admission to the general hospitals. Such an establishment has been open for some years in Fitzroy-square; the patients employing their own doctor, and paying three guineas a week which includes everything except doctors' fees; and there are other similar institutions.

### UNEQUAL DISTRIBUTION OF HOSPITALS.

Evidence was given showing in detail the congestion of hospitals and dispensaries in some parts of London, and their comparative scarcity in other parts. Within a radius of a mile from the Middlesex Hospital, for example, there are stated to be eight general and twenty-six special hospitals, with an aggregate of about 2,050 beds, and seven general and six special dispensaries; all those being in addition to the provision made for the sick poor under the poor law.† All the hospitals in London, with very few exceptions, are said to lie within an area of about two miles square.

On the south side of the river St. Thomas's and Guy's are the only general hospitals, neither of which is at present open to its full extent for patients (the Miller Memorial at Greenwich is the nucleus of a third); and the deficiency of hospital accommodation for that part of London was strongly insisted on.

Again, to the east of the London Hospital, in Whitechapel Road, there is great want of accommodation for the sick poor.

If Blackfriars Bridge is taken as a central point, it is said that there are fifty-one hospitals to the west and fifteen to the east (the minor special hospitals being left out of account). Again, a very large district in the north-west is served practically by a single hospital, St. Mary's. The West London Hospital at Hammersmith supplies a very large district, and

* It was said by one witness that five out of the eleven hospitals with medical schools now admit paying-patients (Burdett, 25,849), and the pay system is said to be on the increase (25,842, 25,849).

† The Marylebone Infirmary, situate at Notting Hill, contains 700 beds.

is more than three miles distant from St. George's and St. Mary's, which are the nearest general hospitals. The region about Soho Square is the centre of a great number of special hospitals.

Six miles was estimated as the outside distance in London which an accident case might have to be carried to a hospital.

One effect of the congestion of hospitals in central London was said by one witness to be to annihilate private practice in that district.

### PROSPOSAL TO TRANSPLANT HOSPITALS.

The prevailing, though not unanimous opinion, as appearing from the evidence, seems to be that on the whole the hospital accommodation in London is sufficient* but that much inconvenience and a partial inability in some parts to cope with the demands for admission are caused by the unequal distribution of the hospitals, and by want of organisation. Some witnesses thought the difficulty might be met by the transplanting of some of the hospitals in the central district to places in the north, south, and east, where they are more wanted. Sir E. H. Currie was inclined to favour the Paris system of a bureau central, which should draft off patients when beds were vacant.

It can hardly be doubted that a more equal distribution of hospital accommodation is needed; but at the same time it was pointed out that in settling the position of a hospital some consideration must be shown for the convenience of the medical men who will form its staff; and (though there was evidence in favour of the view that this difficulty could be surmounted) that a hospital in any outlying district would have a difficulty in getting the amount of attendance from distinguished doctors which the chief London hospitals now enjoy. The same difficulty might be found in obtaining a good committee of management, many of the most useful members of such committees being men having business of their own, who could not conveniently attend at great distances.† It was also urged that it would not be so easy to obtain support from public contributions for a very remote hospital. As regards in-patients (except accidents) it appears that the proximity of the hospital to their homes is not generally a matter of such great importance; and, as a matter of fact, it was shown that considerable numbers of out-patients as well as in-patients are in the habit of seeking treatment at hospital remote from their own homes, often passing by the nearer ones and going on to those farther off.

### Proposal to Remove Hospitals to Country.

Another suggestion, involving the difficulties already mentioned, and also difficulties with regard to the requirements of the medical schools, was that a large portion of the establishment of hospitals, including the students, should be removed into the country, only the out-patient department and a sufficient number of beds for accidents and critical cases which would not bear removal, being retained in town. It was urged that at a distance 10 or 15 miles out of town the patients would have a much better chance of recovery than in the vitiated air of London. Although the idea that the London hospitals should have a subsidiary country establishment met with some favour, the general opinion of the witnesses was that the main part of the hospital establishments, including the schools, must remain in town. The immense practical difficulty of altering the existing distribution of hospitals, added to the objections mentioned, seemed to some witnesses to offer insuperable obstacles to any comprehensive scheme of transplantation from one part of London to another, or removal to the country.

### Objection to Large Hospitals.

The late Sir Morell Mackenzie expressed a decided opinion adverse to very large hospitals, which he thought extremely prone to become unhealthy; he even thought it would be an improvement if hospital buildings could be of a temporary character, and be pulled down and rebuilt every ten or twenty years. That very large hospitals are in themselves undesirable was a view which met with a good deal of support, especially where they are planted, as the London Hospital and others are, in the midst of a dense population. Two hundred was mentioned as the maximum number of beds desirable. Mr. Tait considered that with a very large number of beds good management became more difficult, and the death-rate higher, and he gave statistics in support of the latter statement. On the other hand, a witness from the London Hospital spoke in favour of the practical advantages of large hospitals, and in particular of their great value for teaching purposes.

### "Out-Post" Hospitals.

A way of overcoming the inconveniences caused by the unequal distribution of the hospitals was suggested in the establishment of what were called "out-post" hospitals, following the example set by the Seamen's Hospital at Greenwich, which has set up branch establishments in places where sailors congregate; and it was urged that those general hospitals which had surplus funds should apply a part of them in developing this system. The Secretary of the Charity Organisation Society, when questioned on this subject, thought the plan a good one, but was afraid there might be difficulties in the way of its adoption. One witness testified to the usefulness of the Charity Organisation Society as an intermediary between hospitals and poor law.

### WANT OF CO-OPERATION.

Many witnesses drew attention to the want of co-operation among the hospitals themselves and between them and the dispensaries, the poor-law infirmaries, and the privatr practitioners, and various remedies were suggested. So fae from there being at the present time any general system of combination, or any definite division of work among the various institutions, they are on the contrary for the most part competing with one another at every point for public support, and to a great extent for patients. This condition of things is shown to be wasteful as regards subscriptions of the public, and prejudicial, not only to the public who subscribe their money and to the sick for whom these institutions exist, but also to the interests of medical science and education, since a wide field for observation and practice is closed to the clinical teacher and his pupils, while the hospitals, for the sake of their schools, lest the requisite material should fail, are driven to take in and treat a crowd of patients unsuitable for hospital treatment, and the general practitioner complains that he is being ruined.

### Affiliation of Special to General Hospitals.

It was thought that much improvement might be effected by affiliating special to general hospitals, but next to nothing seems to have been done towards bringing about any co-operation between them, except here and there, where a medical man being on the staff both of a general and of a special hospital has transferred a patient of his own from the one to the other. A case of co-operation is that of the Charing Cross Hospital, which sends its eye cases to the Westminster Ophthalmic Hospital; and it was said that cases were interchanged between the general hospitals and the Brompton Consumption Hospital. One witness thought it would not be practicable to affiliate hospitals with infirmaries, and gave various reasons for this opinion.

### Proposal to Form Hospital Districts.

In connection with this system of co-operation, a scheme was proposed, and met with the approval of several witnesses, for dividing the whole of London into districts, each district to be supplied within its own limits with the necessary provision of hospitals and dispensaries, the latter (both voluntary and poor law) being affiliated to the hospitals, and working in co-operation with them. If it proved to be impossible to transplant some of the existing hospitals, and thus make each district self-supporting and self-contained, it was suggested that means might still be found to work the system by attaching territorial areas to the hospitals in their present position. Such a scheme, however, it was thought, could only be carried through after the establishment, and with the advice and assistance, of some controlling body or central board.

* Dr. Bridges, of the Local Government Board, thought the medical relief in London inadequate.

† The want of capable men on their committees was declared by one witness to be the great weakness of the London hospitals (Burdett, 25,653, 25,739-40). The difficulty of getting good men to serve was said to be increasing (Buxton, 8,809).

### HOSPITAL EXPENDITURE AND ACCOUNTS.

### Advantage of Uniformity in Accounts.

The question was asked of a great number of witnesses whether the introduction of a more uniform system of accounts would be advantageous, and was answered almost unanimously in the affirmative. Under the existing circumstances, each hospital making out its own financial statement after its own fashion, it is impossible to form anything approaching a trustworthy estimate of the comparative cost of management and maintenance as between different hospitals. The estimated annual cost of a bed, which is the ordinary standard of comparison, is calculated after so many different methods, producing such widely different results, as to be altogether fallacious. Any such comparison must always be deceptive, unless full consideration is given to, and full allowance made for, the peculiar circumstances of different hospitals, the particular cases and phases of disease which they treat, and the varying cost of the treatment. But in the interests of economy and good management it was strongly represented that an attempt should be made to introduce such a system as should ensure that all calculations of the cost per bed should at least be made upon a uniform basis. Such a reform would assist both the hospitals themselves in checking their own expenditure and the subscribers in judging how their money was spent.

### Cost of Beds.

Attempts have been made to form an estimate of the cost of beds in the several hospitals; and the figures given, if their accuracy could be relied on, would indicate great variation in the annual cost, ranging, according to one estimate, from £181 down to £60. The evidence, however, appears clearly to show that all such calculations are rendered altogether untrustworthy by the want of a uniform basis for making them, and without a settled universal system of account-keeping such a basis cannot be found.

The system of calculation adopted for the Dublin hospitals was mentioned as an improvement on anything in London.

The difficulty of estimating the cost of the out-patients is a serious obstacle in the way of correctly calculating the cost per bed. The mode generally adopted is to deduct from the total expenditure a sum calculated on a more or less arbitrary basis at from 1s. to 2s. or even more for each out-patient; but it is found impracticable to keep the expenses actually incurred for the out-patients distinct from the general expenditure. To do so it would be necessary, amongst other things, to incur the additional expense of keeping separate dispensary accounts, and perhaps separate dispensaries.

Attention was drawn to the difference of cost per bed between the hospitals and the poor law infirmaries. This appears to be accounted for by the difference in the numbers of the medical and nursing staff, and in the character of the cases treated; the chronic cases, which form the majority in the infirmaries, requiring less expensive treatment and less nursing than the acute cases in the hospitals.

It was complained that the expenditure for particular purposes, such as stimulants for the use of patients, could not in all cases be ascertained.

### Glossary or Index of Classification.

The Secretary of the Seamen's Hospital at Greenwich had worked out, and he described, in detail, a model system of accounts by which an effective comparison could be made between different institutions. The important point in his proposal was that there should be not only a uniform basis of account, but also a somewhat minute sub-division of the heads of expenditure, and a glossary* showing exactly what items were to be included under each head. Without such a glossary no form of account could, it was said, be really trustworthy for purposes of comparison; such things as mineral waters and condensed meat-juice, for example, would appear sometimes under "provisions" and sometimes under "dispensary," and a host of minor discrepencies of this kind would inevitably lead to erroneous inferences. This glossary system was supported on the ground that it would enable the governing body of each hospital to keep a very close control on each item of expense by comparing it with the same item elsewhere, and that it would promote inter-communication and exchange of experience between hospitals. It met with some (but not universal) favour from other witnesses.

### Progress to Uniformity Promoted by Sunday Fund.

Some progress towards a more uniform system of accounts has already been made through the action of the Hospital Sunday Fund, one of whose objects it is to effect this reform. No hospital is qualified to receive a grant from the fund unless it furnishes a statement of its accounts in the prescribed form. The form, however, is framed merely to meet the requirements of the administration of the Fund, and does not supply the particulars required for a complete comparison, in detail, of the cost of hospital management. For example, it distinguishes between "proprietary" and "charitable" revenue, and shows the amount contributed by patients; the object being to arrive at the sum representing the annual "needs" of the hospital from the public. Then there is a division between expenditure for "maintenance" and that for "management"; this is for the purpose of ascertaining whether the hospital is economically or extravagantly managed.

It was hoped that through the Sunday Fund further advances would be made towards uniformity; but objection was taken to any attempt being made at forcing all the hospitals into an exact method; this, it was thought, savoured too much of State control, and would tend to destroy individuality.

The proposals which were made regarding the establishment of some form of central body, with a limited control over hospital administration, included generally the vesting in such body of the supervision of accounts.

### Effect of Medical School.

Some evidence was taken as to the effect which a medical school has upon the finances of the hospital to which it is attached. Some witnesses thought that the school must be indirectly a source of expense to the charity, because it rendered necessary the early and experimental adoption of scientific improvements and appliances which, without it, might have been dispensed with, and that, therefore, the medical schools were partly supported by charity; but there was rebutting evidence on this point, and it would seem that expenditure of this kind must, to some extent, be a direct gain to the patients, and, therefore, may be properly defrayed to that extent out of charitable funds; while, at the same time, the students gratuitously render services which could not otherwise be obtained without expense. At the Charing Cross Hospital, the school makes a fixed contribution to the general funds of the hospital, and the hospital appears to make a small net profit. Expenditure incurred for enlarging the school was said to be there regarded as an investment. One witness, while he was of opinion that the school undoubtedly increased the expenses, thought that it also greatly increased the income of a hospital by widening thearea of public interest and support.

### FOOD IN HOSPITALS.

Evidence was taken respecting the general treatment of in-patients. the regard shown to their comfort, the means available to them of making known their complaints, and in particular respecting the quality of the food supplied to them. Upon this latter point a great number of questions were asked, but, on the whole, little evidence was elicited of an unfavourable character. One witness, indeed, considered that the hospitals were administered, in matters concerning the comfort of the patients, on an unnecessarily luxurious scale. The defects which were mentioned were not of a very serious or deeply-rooted character; and strong evidence in confirmation of the general good administration of the hospitals in all that concerns the comfort of their patients was given by the chairman of the Saturday Fund, who (as is mentioned above) testified that the great majority of complaints which had been brought to his notice by ex-patients had proved, on investigation, to be unfounded. The patient appears generally to have sufficient opportunity of complaining of anything wrong, both to the nurse, who is specially charged with his care, comfort, and diet, and also to the visitors who, in most (but not all) hospitals, are specially appointed to go round the wards and inspect everything, and investigate complaints.

At many hospitals it is the practice to require patients to provide their own tea and in some cases butter; and it was

* Published in Burdett's "Hospital Annual."

said that at one hospital all the tea was mixed up, and the mixture was not good.

The usual system in the large hospitals appears to be that the sister of each ward makes up a diet sheet for the day, in accordance with the doctor's directions for each patient; the steward (or official charged with this duty) has to provide the food and get it prepared and served up. Then it is the duty of the "sister," who is usually the head nurse of the ward, to see that the meals actually supplied are in accordance with the diet sheets.

At one hospital it is the custom for the chairman to see every patient on leaving, and ask him if his food has been good.

### NURSING.

The great improvement in hospital nursing of recent years was testified to by several witnesses.

The nursing staff of a hospital ordinarily consists of a matron or lady superintendent, a certain number of head nurses, usually styled "sisters," one to each ward or pair of wards (according to their size) by day, and one for the whole hospital, or a wing of it, or for a group of wards, by night; staff nurses, that is to say, nurses who have passed their full period of training and received their certificate; and probationers, these latter forming the most numerous class. The more advanced probationers are often entrusted with the duties of staff nurses. In addition to the ordinary probationers there is, in some hospitals, a class of paying probationers or lady pupils, who perform the same duties as the others, but whose terms of service are different.

### Probationers.

The probationers are engaged by the matron, subject or not (according to the rules of the particular hospital) to the sanction of a higher authority, or are enaged by the hospital authority on her recommendation. The selection rests in all cases, practically, with the matron, and the minimum age at which they are taken is usually about 23. There is no lack of candidates for employment; at the London Hospital, for example, the number of applicants in a single year was said to be 1,600. Nurses are drawn from a well-educated class; many are daughters of professional men, merchants, farmers, and tradesmen. The terms of service differ in different hospitals. But the general rule, as regards an ordinary probationer, is that she is first taken for a month on trial, without wages; at the end of that time, if she is considered suitable and wishes to remain, she enters into a regular contract of service for a stated period of one, two, or three years; during that period, or part of it, she not only assists in the practical work of nursing in the wards, but also attends lectures which are given by the matron or by members of the medical staff, and is required, or encouraged,* to pass examinations; and at the end of the period, having passed her examinations, she receives from the hospital a nurse's certificate.

### Period of Training.

Different opinions are held as to the length of training requisite before a woman should be sent out with a certificate as a trained nurse. A witness who had had experience as matron of St. Bartholomew's Hospital was of opinion that nothing less than three years should be taken as the qualifying period, and that no woman ought to be made sister of a ward or staff nurse, or be sent out to nurse the sick until she had passed through the whole curriculum.† Miss Nightingale, on the other hand, has laid down one year as the ordinary period of training, with the proviso that it would be preferable to give two years' training to those who will have to train others in their turn. At St. Thomas's, where the nursing is organised according to Miss Nightingale's system, the probationer, after her month's trial, binds herself to hospital service for four years; after one year, if she passes her examination, she is registered as a certificated nurse, and thereupon for another three years she holds herself at the disposition of the Committee of the Nightingale Fund for hospital nursing. At other hospitals the engagement does not extend beyond the period of training, but that period is prolonged to two or three years, so that the hospital, after it has trained the nurse, may still have the benefit, for a time, of her trained services; the longer period being fixed rather for the sake of increasing the nurse's experience, and for the convenience of the hospital, than from the belief that she would not be fit to receive a certificate sooner. At the London Hospital, for example, a nurse is certificated after two years' service, but is in some cases given the duty of a fully qualified nurse in the hospital, or sent out to nurse a private case, occasionally is even appointed to be a sister of a ward, while still called a probationer. Length of service is only one of several elements which go to make a good nurse; and the opinion was strongly expressed that more reliance was to be placed on a system of careful individual supervision and selection than on any extension of the probationary period. At the London Hospital, out of about 210 sisters, nurses, and probationers, fully one half (including about 50 probationers in the second year) were regarded as qualified nurses.

At St. Bartholomew's, the certificate is given after three years, and a gold medal to the best nurse. A probationer having passed an examination after one year, is called a staff probationer, and may be employed as a staff nurse. At Guy's, the probationer, after her month's trial, seems to be taken on for a year, and then (if she gives satisfaction) for a further term of two years; at the end of the three years she gets her certificate, but she becomes a full nurse (though uncertificated) after 18 months, and is then qualified to enter the private nursing institution.

At St. George's and the Middlesex the certificate is given after three years, but the probationer is promoted to be a ward nurse after one year. At the Middlesex it was not until recently the practice to send out a nurse for private nursing before she had been five years in the hospital; but exceptions are now made to this rule, and nurses are in some cases allowed to go out after three years' training.

At Charing Cross the period is three years, at St. Mary's two years, at the Royal Free three years, at Brompton three years, at King's College three years, but after two years the probationer generally becomes a staff nurse; at the Homœpathic Hospital three years, but a nurse is considered to be trained after one year.

### Paying Probationers.

The paying or special probationers, or lady pupils, who are taken at some, but not at all hospitals, usually enter for a three months or other short course of training;* but sometimes they remain for a second course, or they become ordinary probationers. The usual payment made by them is at the rate of a guinea a week. At some hospitals they are separately lodged, but their duties seem generally to be the same as those of the ordinary probationers.

### Sisters.

The appointment of the sisters rests with the executive authority of the hospital. They are in a position of considerable responsibility, each having, under the matron, the entire charge of her ward; and at some hospitals they are generally selected from among nurses of superior social position. It is the matron's duty to make frequent visits to the wards. In most hospitals she appears to go round daily; but whether she does so or not the sisters are fully responsible to her for the state of their wards and the proper fulfilment by the nurses of all their duties; and they have the immediate superintendence of the training of the probationers. Nurses are not usually taken over the age of 35.

Each sister usually sleeps in a room adjoining her ward, so that she can readily be summoned at night in emergency.

### Ward Maids.

The work of the nurses is supplemented by ward maids and scrubbers. The ward maids sometimes but not always are lodged in the hospital and some of the smaller hospitals have no separate class of ward maids. Inquiry was frequently made whether the nurses were called on to perform menial duties. The rule seems to be that it is their business to do everything directly affecting the patients, including a good deal of sweeping and dusting; they also generally clean the lamps, and sometimes inkstands; in one case, it appeared that a portion of the floor was polished by probationers, but this was quite an exceptional case. The

* At the London Hospital the examinations are not compulsory, but a nurse who has passed a satisfactory examination has a different form of certificate.

† It is, however, to be noticed that St. Bartholomew's has only 20 certificated nurses to 141 probationers—a fact which appears to indicate that a large proportion of the probationers are considered to be fully qualified nurses.

* The lady pupils at Guy's undertake to remain for a year, and at the Middlesex for a year or six months. At St. Mary's they enter for one or two years, and pay £30 a year; at the Seamen's Hospital the payment is £25 a year.

evidence generally was to the effect that the nurses were called on to perform a certain amount, but not a great deal, of work which did not properly belong to their office. Some of the Matrons would gladly see an addition to the number of ward maids.

A probationer, during her first year, is paid usually at the rate of about £1 a month, or rather less; after that she rises to £18 or £20 a year, but in some hospitals no salary is given during the first year. The pay of fully trained nurses in the hospitals, and in the private nursing institutions attached to hospitals, ranges from £20 to £35 or £40;* those employed for private nursing being as a rule (as already mentioned), much better paid than those in the hospital, who do not generally rise so high as £30†. The night nurses get rather more than the day nurses. Sisters usually receive from £35 or £40 to £50 or £60. Sometimes the rate of pay is rather lower than the above, and a gratuity or pension, or both, are allowed by the hospital after a certain period of service; and the institution nurses are sometimes allowed a percentage on their earnings. Board, lodging, and often some articles of clothing are provided free, but not, as a rule, washing. The grant of an allowance of 2s. 6d. a week for washing is one of the reforms suggested.

### Proportion of Nurses to Patients.

Roughly speaking, at the present time, if the whole nursing establishment is to the total number of occupied beds in the ratio of 1 to 3½, it is considered a fairly high proportion. If that test is applied to a few of the leading hospitals the following results appear:

St. Bartholomew's, about 200 nurses; average number of occupied beds, 570 out of 667; about 1 to 3.

St. Thomas's, 117 nurses; about 436 patients (not including the paying ward); about 1 to 3¾.

London, 218 nurses; maximum number of occupied beds, 733; about 1 to 3½.

Middlesex, 88 nurses; average number of occupied beds, nearly 260; about 1 to 3.

Charing Cross, 51 nurses; 165 occupied beds; about 1 to 3¼.

St. Mary's, 61 nurses; 255 occupied beds; 1 to 4 1·5.‡

King's College, 78 nurses; maximum number of occupied beds, about 215; 1 to 2¾.

Westminster, 55 nurses; maximum number of occupied beds, about 200; 1 to 3⅝.

University College, 80 nurses; maximum number of occupied beds, about 200; 1 to 2½.

Royal Free; 1 to 3½.

Whether the proportion of nurses to patients be considered sufficient or not, there can be no doubt that it has in recent years been very materially increased; thus it appears that in 1880, at the London Hospital, it was 1 to 5; and the staff at St. Bartholomew's is said to have doubled in the last 10 years.

A statement read by the Matron at the London Hospital showed that on a given day in the summer of 1890 the number of patients was 626; and the number of the nursing staff actually on duty was 124 on day duty and 55 on night duty, giving on the whole about 1 nurse to 3½ patients. The same witness was of opinion that if money were no object the proper staff actually on duty in a ward of 30 beds would be a Sister, two staff nurses, and two probationers by day, and a staff nurse and two probationers by night; she thought there should also be three ward maids to two wards. The late Matron of St. Bartholomew's would add another probationer for day duty, making the total number by day six instead of five. In the children's wards the proportion of nurses should be higher. In addition to this, which would be the normal staff on duty, a margin of strength would have to be provided for the cases requiring special nurses.§

The figures given above (as well as other evidence to the same effect) appear to show that while the strength of the nursing staff on duty by day at one of the great hospitals (the London being taken as an example) is fully sufficient for the needs of the sick, by night the strength is somewhat short,* the deficiency being however such as would be remedied by a trifling increase in the number of nurses on the establishment. The demand for an increase in the nursing staff is in fact made in the interest rather of the nurses themselves, in the interest of shorter hours of duty and longer holidays, than of the patients. There was little evidence that patients suffered from insufficient nursing, while on the contrary abundant testimony was forthcoming of the admirable care and attention bestowed on them, and of the spirit of self-sacrificing zeal which animated the nurses.

### Hours of Duty.

The following appears to be the average daily routine, but each hospital has its own scheme of service, and allowance must, therefore, be made for variations in detail:—

The day nurses come on duty at 7 a.m.,† having breakfasted at 6.30 or 6.45. The sisters in some hospitals come on an hour later.‡ The first hours are busily occupied in getting the patients fed and washed, their beds made, and the wards put in order for the day. Later (both before and sometimes after dinner), the doctors have to be accompanied on their rounds, and the orders for the diet, medicine, and general treatment of each patient carefully noted; but this is rather the work of the sister than of the subordinate nurses. A short time is allowed in the course of the morning for getting some luncheon; and half-an-hour, sometimes a little more, but at some hospitals it seems a bare half-hour or less, is allowed for dinner, the sisters and nurses going generally in one relay, and the probationers in another.

The nurses sometimes take their tea away from the wards, and they go off duty at 9 p.m., at some hospitals not till half-past 9 or 10.

The night staff breakfast at 8.30 p.m., and come on at 9 p.m.,§ and remain till 9 a.m., or a little later; there are thus 2 hours in the morning when both the day and night nurses are on duty together, those being the busiest hours in the day. During the night the nurses have two meals, either in the wards or in the kitchen. At some hospitals a point is made of their going twice for proper meals away from the ward.

The full hours of service are thus 14 or 15 hours for the day nurses, and 11 or 12 for the night nurses. From these hours certain deductions have to be made, both for meals and for time allowed off duty. The average allowance to the day staff is, for meals, from 1 hour to 1½; and in addition each nurse will, under ordinary circumstances, be allowed a certain time, varying from day to day, for exercise and recreation. At St. Bartholomew's, for example, it appears that the sisters are off duty from 6 p.m. to 9 p.m. every other day, from 2 p.m. to 10 p.m. once in two weeks, and from 3 p.m. to 9 p.m. every alternate Sunday; they are also free once a month from 4 p.m. on Saturday till noon on the following Monday. The staff nurses have a rota of four weeks; in the first week they are off duty from 6 p.m. to 8.45 p.m. on two days; in the second week, once from 6 p.m. to 8.45 p.m., and once from 2 p.m. to 9·45 p.m.; the third week is like the first; and in the fourth week they are off a whole day to 9.45 p.m., and have also one evening off. Practically, the actual hours of duty at St. Bartholomew's were said to be about 11, an hour being allowed out of the fourteen for meals, and two hours on the average off duty besides.

At St. Thomas's a full day's work was said to be 10 hours actually on duty, rarely more; but the average number of hours per week would not be more than 61, allowance being made for half a day off during the week, and four and nine hours on alternate Sundays.‖ At this hospital the nurses are said to be especially well off.

At St. George's the head nurses are on duty from 7 a.m. till 10 p.m., with two hours off, besides meal times, and one

* At the Royal Free Hospital an institution nurse, after four years, receives £30 salary and £20 bonus every year.

† At King's College Hospital, and at the Fever Hospital, a nurse rises to £36.

‡ St. Mary's obtains additional nurses, when required, from the Brompton Hospital; but the proportion of nurses seems to be somewhat low at St. Mary's, as there is said to be one nurse to seven patients by day, and two to 43 patients by night.

§ The medical superintendent at Guy's spoke of one day nurse to 12 patients, and one night nurse to 20 patients as being about a fair average proportion for the actual work of attending to the patients in ordinary cases.

* Confirmatory evidence, as to the night staff being shorthanded, was given from St. Bartholomew's.

† At Guy's, 8 a.m.

‡ And sometimes go off later. At the Middlesex the sisters are on duty till 11 p.m. At St. Bartholomew's it is said there is no definite hour at which the Sisters go off.

§ At Guy's the night nurses are on duty from 9.30 p.m. to 8.30 a.m.; at St. Thomas's, from 10 p.m. to 9 a.m.

‖ Another witness, however, estimated that at St. Thomas's the sisters and staff nurses worked alternately 73 hours and 79 hours per week; probationers 65 hours in the wards.

whole day and one half-day once a month. The other day nurses go off duty on alternate days at 6.45 and 9.30; and they have one day off in a month.

At the Middlesex the sisters are said to be actually on duty for 11 hours and the nurses for 10 hours, but it would seem to be longer than that on some days. The sisters have a whole day every month, and the nurses every alternate month.

At Charing Cross the sisters are said to have 58 hours a week on duty, and the nurses 67½ hours.

At St. Mary's the average hours of actual duty are said to be 10⅕ hours for a sister, 9⅔ for a staff nurse, and 9½ for a probationer.

At King's College the hours appear to average about nine.

The ex-matron of St. Bartholomew's thought that every nurse ought to have half a day off duty every week and three hours off every day.

The night nurses, with the exception of the time for meals, are on duty during the whole 11 or 12 hours, but it is explained that their duties, as compared with those of the day nurses, are generally less onerous and involve less moving about and standing. This, however, does not appear to be universally true; as there was evidence that in some hospitals, where the wards are small and the night staff weak, the nurses are obliged to keep moving about continuously from ward to ward during the whole night.

It was explained that at the London Hospital each nurse has a book in which a detailed record is kept of what she does—work, day or night duty, sickness, holidays, &c.

Holidays.

The length of holiday allowed during the year varies from a fortnight to a month, except at Guy's, where the sisters have one month in summer, and a week or ten days at Christmas. It was the opinion of several witnesses that three weeks was the shortest time to which nurses should be entitled; some witnesses thought that the sisters, in consequence of the more responsible character of their duties, required a longer holiday than the ordinary nurses.

The matron of the London Hospital advocated a month's holiday for all nurses, and six weeks for the sisters.

Arrangements are generally made for the holidays to be taken during the summer months.

Food.

Evidence touching the question of the food provided for nurses was noticed in connection with the London Hospital. On the whole the improvement in this respect seems to have kept pace with the general progress of reform. Matters of complaint were mentioned; but they appear, for the most part, to have belonged to a past time. At all events, the importance of a superior diet for the nurses, in view of the character of the work required of them, is everywhere recognised. Some criticisms were passed on the system of allowing some of the meals to be taken in the wards.

Alleged Overworking of Nurses.

Several witnesses expressed the opinion that the existing hours of duty for the nurses were too long, and the labour unduly arduous. Out of the ten or eleven hours on duty, it was estimated that a nurse would generally be actually on foot for about nine, and nurses are peculiarly liable to be afflicted with flat feet owing to the excessive amount of standing and moving.

If, however, the question of health be taken as a test whether nurses are overworked or not, it cannot be said that the evidence proved conclusively any general inability to stand the strain imposed by the existing conditions of nursing. The proportion of nurses who break down from bodily weakness or too great nervous sensibility does not seem to be large; the reports made from the various hospitals were generally favourable as regards the health of the nurses, and the opinion was several times expressed that they were not overworked. A lady at the head of the nursing staff of one hospital held that women gave up ten years of their lives by entering this profession, but that view was altogether rejected by others.

Improvement in Position of Nurses Hoped For.

Mr. Rathbone's opinion regarding the necessity of increasing the nursing staff of hospitals was that "the patients are our first objects in hospitals, and if hospital work is such work that a woman of ordinary health and strength can do it and remain in health, . . . . I think you then have done all that you are bound to do until the public gives you money to do more." This opinion, that a further relaxation of the labour required from nurses was a matter of money and of comfort, rather than of necessity either to the nurses themselves or to the patients, was the opinion of more than one witness from within the hospitals. At the same time, even those who considered the nursing staff at their own particular hospitals to be numerous enough for their duties, regard being had to the wants of the patients and to all existing standards of adequacy, were hopeful that the position of nurses generally would in the future be improved by means of shorter hours of labour, longer holidays, and better pay.

The want of accommodation for more nurses forms in many hospitals an obstacle to increasing the staff.

### THE COMMITTEE'S CONCLUSIONS.

Endowed Hospitals.

The Committee observe that only when the endowed hospitals wish to deal with their estates, or to alter the fundamental conditions on which they administer charity, can the Charity Commissioners effectually intervene. The practice is that the endowed hospitals send their accounts annually to the Charity Commissioners; but the action of the Commissioners is limited to receiving these accounts, and the Committee recommend that the Commissioners should have power to audit the accounts, and to see that the endowments are applied according to the trust.

For the building of St. Thomas's Hospital the authorities had to borrow £100,000 at the rate of 4 per cent., which was afterwards reduced to 3 per cent. St. Thomas's Hospital has £27,000 invested with the Charity Commissioners, and the Committee consider that it is to be regretted that by the action of the Charity Commissioners the hospital was prevented from using its own money.

In the case of the three endowed hospitals, the Committee are of opinion that the system of administration does not on some points compare favourably with that which exists at the other general hospitals. It throws too much power and responsibility into the hands of one individual, the treasurer; though at St. Thomas's Hospital a larger share in the administration is assigned to committees than at the other two. The Committee would especially direct attention to a report by Dr. Thorne (see paragraph 18 of this report), showing that the nursing home of St. Bartholomew's Hospital was in a very unhealthy state, to such an extent that 23 nurses and three ward maids were attacked with diphtheria; and also that the drainage arrangements of the three principal ward blocks of the hospital square were defective, and "could not be too strongly condemned." The Committee consider that, had there been a large committee of governors alive to the responsibilities of their office, such a discreditable state of things would not have been allowed to occur. It appears in the evidence that the surveyor, a salaried officer, during the three years that he had been in office, had never been called upon to make a thorough examination of the drainage of the hospital; and though a report was at length made, it was not a thorough report, the excuse being given that it had to be ready by a certain date, and that there was not time to make it as thorough as it ought to have been. This neglect is, in the opinion of the Committee, the more inexcusable owing to the affluent circumstances of this charity.

The Committee would suggest that in all these endowed hospitals the government should be carried on by a system of weekly boards and sub-committees.

As regards St. Thomas's and Guy's, the Committee greatly regret to remark that owing to want of funds, occasioned by fall of values, for the most part in agricultural rents, a certain number of beds are obliged to be kept vacant in each hospital, while others are let to paying patients.

Eight General Hospitals with Schools.

The remaining eight general hospitals with schools depend entirely for their support upon voluntary contributions, excepting in a few cases where they possess some small endowment.

Their systems of management greatly resemble one another, and the evidence shows that they are generally well administered. The Committee note the enormous amount of work done by unpaid boards of managers; and the care exercised, as far as the Committee are able to judge, in the appointment of their medical as well as other officers.

The Committee desire to refer to the personal nursing dispute appearing in the evidence of the London Hospital. The authors of these charges were for some time nurses and probationers in this hospital, some of whom did not remain during the whole period of training, and of whom two, at least, stated grievances of their own which were not confirmed by the evidence; and the late chaplain who, for some time before the termination of his connection in that capacity with the hospital, had differences with the Committee both in these matters and also in regard to the performance of his own duties.

The charges are, on the whole, in the opinion of the Committee, not substantiated by the evidence. The evidence in regard to the injury to the health of the "Sisters" appears inconclusive. The Committee consider that the difficulties would have been avoided had the governing board, in charge of the hospital at that time, not allowed their authority to fall into the hands of salaried officers. In justice, however, to the London Hospital, the Committee wish to add that it is an admirable hospital, doing work in a part of London where it confers inestimable benefits upon a very large and very poor population. They, therefore, think it is deserving of the greatest measure of charitable support.

The Committee recognise that it is advisable, under present circumstances, to maintain the individuality of these general hospitals, and they consider that the generous rivalry thus promoted tends to medical and administrative efficiency.

The Committee suggest that the fact of not holding the diplomas of the Royal College of Physicians and Royal College of Surgeons of London, should not exclude practitioners who have graduated elsewhere from becoming members of the staffs of the general hospitals in London. At present at only one general hospital, St. Mary's, are there no restrictions. The Committee would gladly see the restrictions removed at the other hospitals in London.

### CONVALESCENT HOMES.

The Committee remark that the accommodation for convalescents in connection with the large hospitals is insufficient, only two or three having convalescent homes attached to them; and that this want is met by the authorities of the hospitals subscribing, through the Samaritan Fund, to convalescent homes.

Owing to the scarcity of the accommodation the patients, although not thoroughly cured, are discharged, if well enough to leave the hospital. In some cases these patients find their way to the Poor Law infirmaries; in other cases, patients suffering from medical complaints have to be kept for long periods in a hospital, although they would recover more rapidly at a convalescent home in the country. Moreover, these patients have to be provided for in the hospital, to the exclusion of those who would be admitted were beds vacant.

The Committee avail themselves of this opportunity to direct attention to this need, in the hope that more extensive convalescent accommodation may be provided by philanthropic effort.

### OUT-PATIENTS AND DISPENSARIES.

The Committee received much evidence on the subject of the out-patient system. On the one hand were set forth the advantages of large out-patient departments for teaching purposes, and for the relief of the poor, as they are open at all times day and night, and the great advantage they afford as centres for consultative purposes. On the other hand, it was urged that unlimited medical relief was the first step towards pauperizing large masses of individuals. The witnesses who held this view pointed out the advantages of provident associations. Those who had not the means to belong to a provident association could obtain medical relief from the institutions provided by the Poor Law.

It was suggested that it might be advisable to map out London into districts; and that a person leaving one district, and therefore the provident medical association, could easily attach himself to the provident medical association of his new district. But the Committee, agreeing that such an arrangement would be highly desirable if it were practicable, doubt whether in London, with its heterogeneous and migratory population, such an organisation would be possible.

It is considered by the Committe that by the abolition of the out-patient departments medical education would be seriously interfered with, and further, that on the whole it must be left to the authorities of the hospitals themselves to arrange the organisation of the out-patient department, with the view of rapidly attending to the requirements of the public, and of insuring as far as they can that the charities shall not be abused.

The Committee are of opinion that the charities are not abused to any serious or appreciable extent, nor do they think that it was by any means proved that patients are carelessly treated, or treated by students instead of thoroughly qualified medical practitioners.

The evidence respecting fees appears to show that above the sphere of the Poor Law there must exist a large section of the population who cannot afford to pay a doctor in the case of long and serious illness, or in the case of a large family.

On reviewing the evidence as to the different systems pursued by the different great general hospitals, the Committee think that, on the whole, the system of limiting the number of out-patients per diem is the most convenient.

The Committee consider that inquiries should be made, wherever experienced officials think there is cause for suspicion, and that the patient should establish a *primâ facie* case for charitable relief.

It was difficult to obtain from witnesses the exact amount of the work of an out-patient department, because the return of new cases only shows about a third of the work done; it was, however, generally agreed that each patient attended on the average about three times. The Committee do not attach too much importance to the statements as to the reduction of fees of practitioners among the poor by the free work of the hospitals, but it is obvious that the existence of the charities must tend to reduce them.

Medical practitioners and the medical officers of free and other dispensaries should be encouraged as much as possible to take advantage of out-patient departments as centres for consultative purposes, and, from the evidence of many hospital witnesses and others, this is already done to a certain extent. In the case of dispensaries and practitioners, the patient might be left in the hands of his medical adviser and not necessarily taken into the hospital.

### DISTRIBUTION OF HOSPITALS.

The Committee observe with regret that on the south side of the Thames there is very little hospital accommodation compared with that on the north side. St. Thomas's Hospital and Guy's Hospital, already shown to be obliged, for want of funds, to close their doors to many of the sick poor, are the only large general hospitals south of the Thames, but they are situated in the extreme margin of the southern district. One witness from the south side described the medical relief as lamentably deficient; at the same time it was stated that Lambeth Infirmary was full.

On the north side of the Thames, especially in the region of Soho, there is great congestion of hospital accommodation. It was stated by a witness that within one mile of the Middlesex Hospital (Berners Street, Oxford Street) there are over 2,050 hospital beds, as well as 13 dispensaries of various kinds; in fact, that by far the greater proportion of the institutions for medical relief are within an area of two miles square. In addition to this local accommodation for the sick, there is the Marylebone Infirmary at Notting Hill, where there is accommodation for 650 patients; Paddington Infirmary, 180 beds; and Central London Sick Asylum, 264.

It was suggested that certain hospitals might be removed from places where they are not so much required to localities where the accommodation is deficient. The Committee cannot regard this suggestion as practical, but they would strongly advise that more hospital accommodation should be provided south of the Thames, and were it possible to find the site, and were philanthropic endeavours to be made for further accommodation for the sick in London, a large general hospital, say in the densely-populated district of Camberwell, would no doubt be of extreme value.

The Committee do not lose sight of the tendency of individuals to prefer some particular hospital, and many instances were given of patients passing four or five hospitals on their way from their homes to a particular hospital in which they had confidence. Though the Committee cannot doubt that this is a fact, and that possibly this migratory disposition would not be checked by the building of a large general hospital, they are nevertheless convinced that more hospital accommodation is required south of the Thames.

### Education.

The Committee had before them all the deans of the medical schools, and heard opinions from some prominent members of the profession. Many witnesses put forward views in favour of and against central colleges for the teaching of certain subjects. The Committee consider it well worthy of consideration whether it would not be advantageous that the medical schools in London should affiliate themselves to a teaching university or organisation, after the nature of colleges in a university, with the view to the securing first-rate lecturers for the subjects which can be taught in classes as distinguished from clinical instruction.

The Committee observe that a very useful field for medical instruction is at present closed to students—namely, the Poor Law infirmaries. It was the opinion of nearly every witness that these infirmaries could be usefully opened for clinical instruction. In this the Committee heartily concur. In addition to the large field for instruction which would thus be opened, they agree with the opinions expressed that the presence of students is to the practitioners stimulating, by reason of the observation and criticism which is brought to bear on diagnosis and treatment; and the evidence they have received shows that where a system of clinical classes of students is carried out under proper regulations the patients have no objection to students at their bedsides.

It appears that there are only three hospitals where female clinical clerks are employed—the Hospital for Children in Great Ormond Street, Royal Free, and New Hospital for Women. Witnesses from these hospitals testify to the ability and address with which the duties of such clerks are performed.

### Special Hospitals.

The case of special hospitals and the arguments urged for and against this class of hospital are summarised in the preceding pages. Hospitals for certain diseases of patients—for example, for children—do not appear to the Committee to be open to the criticisms made on special hospitals.

Lock hospitals form a separate subject for consideration. The Committee think that the nature of the disease and the character of the patients make it desirable that they should be treated in separate buildings, or, at all events, in separate wards from other patients. The Committee have had their attention particularly directed to the fact that patients in these hospitals are in the habit of quitting the hospital in a diseased state on such occasions as the Derby week, fairs, &c., for the purpose of pursuing their avocation. The Committee recommend that provisions analogous to those which prevent a patient leaving a hospital when suffering under infectious diseases should be extended to certain venereal diseases.

Objection is made to special hospitals on the ground that exclusive attention to a particular disease tends to narrow the mind and to induce a specialist to imagine that all complaints are in some measure connected with the disease to which he has devoted so much attention. It is obvious that there is a certain tendency in any special study to narrow the mind, but any such consequence is practically avoided if the practitioner goes through a sufficient course of general hospital and other general practice before he elects to devote himself as a specialist to a particular disease. It is impossible to prevent the natural consequences of the great competition in London to force men into eminence in respect of their special knowledge and familiarity with particular complaints. After all the evidence presented to them it seems to the Committee that the hostility, so widely shown by the medical profession to special hospitals, arises from the fact that numerous small hospitals for special diseases have been instituted by medical men for the purposes of their own advancement, and that such a course of action leads to the establishment of hospitals where they are not wanted, to waste of money incident to the creation of badly-managed and small institutions, and to the deception of the public by inducing them to subscribe to undertakings alleged to be of public benefit, but which are in reality mere schemes for private emolument, and also are useless for teaching purposes.

The Committee consider that the charge of abuse is substantiated in regard to some small special hospitals. This class of small special hospitals to which the Committee refer, of which examples appear in the evidence, the Committee do not consider of any real benefit either to the sick or to science. They appear to be carried on sometimes in incommodious buildings or under unsanitary conditions, and the Committee would deprecate the multiplication of such institutions.

The Committee think it their duty to invite particular attention to the case of the Royal Hospital for Incurables at Putney. While in receipt of very large support, having a surplus in 1889 of £16,000, the authorities of this hospital appear to be incapable of effecting reforms, and are extremely resentful of external observation. In regard to this hospital the Committee would strongly recommend reforms in this direction: That a resident medical officer should be appointed with general control in absence of the Committee, as is the case in the Poor Law infirmaries; that a ladies' Committee should be appointed, as a large majority of the patients are females; that all nurses should be hospital-trained; that the contracts for food, and stores of all kinds, should be by open tender; and that the general supervision by the Committee of Governors should be greatly increased. The objects of this charity are excellent, but until the management is thoroughly reformed the Committee regret that they feel bound to add that the institution is not one which can be commended.

### Accounts.

The Committee observe with satisfaction that, since the opening of this enquiry, a Committee, comprised of the Secretaries of some of the principal London hospitals, has been considering the subject of a uniform basis of accounts, a copy of which appears in Appendix A to the report. The Committee are glad to notice that those best acquainted with hospital accounts have recognised the advisability of a uniform system. The Committee consider that, for accuracy, further sub-division on the expenditure side might be advisable; as for instance, "firing and lighting;" also "wines and spirits," might be tabulated separately. Under heading VI. it might be well to state, for the information of the public, for whom the "salaries, wages, and pensions," as well as "other salaries, wages, and pensions," are charged. It might be worth while for the Committee of hospital Secretaries, if it renews its sittings, to consider whether the totals might be stated on one page, with letters referring to schedules, where the items of expenditure might be set forth in greater detail.

In the evidence before the Committee mention was made of the difficulty of ascertaining the cost of an out-patient without which calculation any estimate of the cost "per bed" is unreliable. The Committee do not think the difficulties insurmountable. The main difficulty appears to be to separate the accounts of the dispensaries into two parts—in-patient and out-patient; this once arranged, the reliable cost per bed might be ascertained. The Committee consider that this difficulty might be met thus—an account might be kept of any drugs supplied for the in-patients; the difference between the total dispensed and the amount supplied to the in-patients would be the amount supplied to the out-patients. The wages of the nurses in the out-patient department, and the wages of the scrubbers, porters, &c., employed could be charged to the out-patient department. The proportion of rates and taxes might be estimated by the proportion which the space allotted to the out-patient department bears to the whole hospital.

### Contracts.

The Committee consider that all contracts should, as far as possible, be by public tender, according to the practice enforced by the local board in regard to Poor Law infirmaries.

### Co-operation.

The Committee regret to remark that there does not seem to be any genuine wish for co-operation between the various kinds of medical institutions. They are of opinion that much more might be done than at present by the hearty co-operation between the special hospitals and general hospitals, between dispensaries of all kinds and general hospitals, and between general practitioners and general hospitals. It would be an early duty of a central board to devise some scheme to further such co-operation.

NURSING.

The subject of nursing is treated at length on pages 73 to 85. A certain amount of variety exists as to the hours of employment of nurses in the general hospitals in London. The Committee consider that eight hours work, exclusive of the time for meals, is, as a rule, as much as should be required from nurses in these hospitals. In constructing future hospitals, care should be taken that sufficient accommodation for nurses be provided to allow of the hours of nursing being reduced.

They would suggest that every nurse in the large and busy hospitals in London should have at least two days off in the month, and that the period of holiday should not be less than three weeks; that not less than one full hour should be allowed for dinner; and while, on the whole, the food of the nurses appears to be good, yet, from the nature of the occupation of nurses, special care ought to be exercised that as well as being sufficient in quantity and quality it should be served in an appetising manner. To bring about this end, the Committee are strongly of opinion that at the nurses' dinner one of the head officials of the hospital should preside, and that the dinners should be frequently visited by members of the governing body.

The Committee note with satisfaction the great preponderence of opinion that the health of nurses in London is good.

The Committee think it very desirable that, where the funds of the hospital permit, pensions should be provided for nurses, whether by the hospital, following the example of the London and Guy's, by joining the National Pension Fund for Nurses, or by the hospital providing a special pension out of its own funds.

Nurses in the wards should not have their duties increased by doing menial work, such as scrubbing and cleaning grates and lavatories, or other services of a like nature. For that purpose, as is the case in most hospitals, the class of servant termed "ward-maids" or scrubbers should be employed.

While the Committee recognise that the Matron must be greatly responsible for the appointment and dismissal and general conduct of the nurses, they are strongly of opinion that no absolute power ought to be given to any Matron, but that the appointments and dismissals should be made by the chief executive authority of the hospital. It is to be observed that many hospitals send out nurses after a certain period of training, at sums varying from one guinea to three guineas a week, to private patients. That these nurses bring considerable addition to the funds of the hospital there can be no doubt. The Committee consider that this is a good practice, but that, to prevent the wards from being denuded of nurses in order to bring funds to the hospital, a separate staff should be employed for this purpose. They are of opinion that the minimum period, after which a nurse can be advertised as thoroughly trained, is three years; and considering the large amount of money these nurses can earn for the hospital, the Committee think that a sliding scale commission on their earnings, mentioned as being in practice at one of the large general hospitals, would be a fair addition to their regular hospital wages.

It appears that at the London Hospital, in the form of certificate for nurses, certain blanks may be filled up in different ways, according to the discretion of the Matron. The nursing capabilities and conduct of the nurse may be described respectively as "excellent" and "exemplary," which constitutes a first-class certificate, or as "good" in both cases when the certificate ranks as second-class. It would seem that the latter form is used when the Matron is by no means satisfied with a nurse; and the Committee think that words indicative of inferiority should be inserted in all certificates below the best, if, indeed, it is desirable that any such certificate should be issued at all.

In regard to male nurses, who appear to be only employed in cases of violent patients, with the exception of two hospitals, every care should be exercised to secure the services, if not of duly-qualified men, of well-known and thoroughly trustworthy persons having, if possible, some experience.

Nursing in the Poor Law infirmaries differs in various institutions. In some a large proportion of nurses are hospital trained; but the Committee regret to find that one-half of the Matrons are not regularly-trained nurses. The Committee are strongly of opinion that not only all Matrons, but that all nurses in a Poor Law infirmary should be trained nurses; the Committee would recommend that no nursing whatever should be done in infirmaries by paupers. The Committee remark that there is no separate infirmary at Bethnal Green, and they observe with surprise and regret that there appears to be in the sick wards in this workhouse a regular staff of less than 20 nurses, some of whom are 65 years of age, and that as many as 80 paupers are employed as nurses.

The Committee consider that the number of nurses should be increased throughout the infirmaries, and that infirmaries should train their own nurses.

This system already exists at one of the largest infirmaries in the metropolis.

POOR LAW INFIRMARIES.

On the whole, the Committee are inclined to think that the system of organisation which places the Resident Superintendent in charge of the whole institution is a good one.

The Committee agree in the suggestion of Miss Twining, that lady inspectors for infirmaries, especially as regards the nursing department, would be a valuable addition to the staff of the Local Government Board.

The new Poor Law infirmaries established since 1867 appear to the Committee, so far as they are able to judge from the evidence, to be well-managed institutions. They think that further accommodation is required, as it was pointed out that a large number of sick poor have to be treated in the sick wards of certain workhouses. The medical supervision is less efficient in the workhouse, while the nursing is altogether inferior. The Committee concur in Dr. Bridge's suggestion that the accommodation in infirmaries should be increased so as to take the patients who are now housed in the workhouses. A notable instance exists in the case of the three unions, the Strand, St. Giles's, and St. James's, which have but a single infirmary between them, the London Central Sick Asylum, containing only 264 beds, as has been observed above, while Bethnal Green has no infirmary whatever. The Committee observe that, although strong representations have already been addressed by the Local Government Board to the guardians of Bethnal Green with a view of increasing their sick accommodation, no steps have yet been taken to remedy the defect; and they are of opinion that if the powers of the Local Government Board are insufficient to enforce a proper provision for the sick they should be extended.

The want of accommodation for the sick is also notable as regards the Whitechapel district, where it appeared that at times the infirmary has 10 per cent. more patients than its proper complement.

HOSPITAL SATURDAY AND SUNDAY FUNDS.

The Committee think the public might subscribe more free to the Hospital Saturday and Sunday Funds could they believe that by these organisations they were really enabled to discriminate between those hospitals which are worthy and those which are unworthy of support.

The system of distributing the Sunday Fund on the principle of "work done," and the Saturday Fund on that of "relief afforded," appears to be open to the objection that it is a premium on competition for patients, and that it tends to stimulate the discharge of patients before the cure is complete, with a view to show as large a return as possible of patients in the year.

PROPOSED CENTRAL BOARD.

Various proposals for a central board are set out on pages 44 to 47 of this report. The Committee do not incline absolutely to any one of these proposals. They are of opinion that, as there is no Government grant, the interference of a Government officer for inspection would be unwise, and they think such interference would tend to check the flow of voluntary contributions, and to some extent to interfere with the responsibility of the unpaid boards of managers.

The Committee do not think that such a central board should be given any statutory powers as regards the formal licensing of any hospital built, or about to be built. They would recommend that the proposed central board should be granted a charter to entitle it to receive endowments, legacies, bequests, and contributions for distribution to medical charities, and to meet its own necessary expenses. The board might be organised in the following way:—

The various hospitals and dispensaries of all kinds should be grouped.

The smaller hospitals should be grouped according to the classes of disease which they treat.

Each general hospital, with or without a school, might be considered to be equivalent to a group.

Each group would send one or more delegates, to be members of the central board.

The heads of the great medical corporations, *e.g.*, the Royal Colleges of Surgeons and Physicians, the Medical Council, and the Society of Apothecaries—might become members of this central board.

The free and part-pay dispensaries might send one member, and the provident dispensaries also one member.

The Hospital Saturday and Sunday Fund might each send one member.

A table (marked "A") is attached, suggesting details for the formation of such a board.

The duties of this board might be of the following nature:—

(1) It should receive annual reports, statements of accounts, and balance-sheets from all hospitals and dispensaries, together with a return of the total number of in-patients, out-patients, and casualty patients.

(2) It should require that all accounts be audited by competent chartered accountants.

(3) It should arrange that all medical charities should be visited and reported on periodically.

(4) It should report from time to time, as occasion required, all proposals for new hospitals.

(5) It should publish an annual report, the principal heads of which might be as follows:—

(A) A complete statement as to the pecuniary position of each medical charity.

(B) A statement by a competent authority as to the existing sanitary condition and ventilation of each hospital, and as to arrangements concerted with the Metropolitan Fire Brigade.

(C) An account of the number of beds in use, the number of beds unoccupied, and the reasons why they are unoccupied. The average daily number of occupied beds, details as to beds for which payment is made, and the number of resident medical staff, resident officers, nurses, and servants.

(D) A statement as to the method according to which each hospital deals with its out-patients and casualty patients, and the number of each.

(E) Proposals for the removal of hospitals and dispensaries to places where further hospital or dispensary accommodation is required, and the proposals for the establishment of new hospitals, and all other matters of interest relating to the treatment of the sick poor.

(F) The nursing at hospitals, and the proceedings of nursing associations in the metropolis.

(6) The proposed board should early turn its attention to the possibility of so organising medical charity as to secure their co-operation with one another, and the co-operation of medical charity with general charity.

While this Board would not have any direct or legal power to stop the building of a new hospital, or to amend systems of organisation in existing institutions, the Committee think that the fear of adverse comment in the reports of the board, or omission from recommendation in those reports, would have a powerful influence in preventing the building of useless hospitals and in securing proper administration in existing institutions.

The Committee think that the Board should assist and work with the managers of the Hospital Saturday and Sunday Funds, and that in addition to the caution which is exercised by the administrators of those funds no grants should be made to any institution whose application was not endorsed by the central body.

There can be little doubt that in times of pecuniary difficulties of any individual hospital or group of hospitals appeals to the public would have greater weight were they supported by a body of responsible men who were conversant with the merits and the means of all the medical charities in London.

The expenses of this Board might be defrayed by levying a small percentage on the income of each group of hospitals sending a delegate to the Board.

In sketching the foregoing outline of a central body, the Committee are desirous of expressing their opinion that some more satisfactory organisation of medical charity is most desirable. It should always be borne in mind that the establishment of Poor-Law infirmaries and rate-supported asylums, under the Metropolitan Poor Law Act, 1867, has in great measure altered the relations between the poor and the hospitals and everything associated with medical charity; and the Committee cannot shut their eyes to the possibility that if some such organisation as they have recommended is not adopted, a time may come when it will be necessary for hospitals to have recourse either to Government aid or municipal subvention.

### TABLE A.

Suggested Grouping of Hospitals for Purposes of Representation on the Proposed Central Board:—

| Group of Hospitals, &c. | Number of Beds. | Number of representatives. | Total representatives of Groups. |
|---|---|---|---|
| 3 Endowed hospitals ......... | 1,912 | 6 | — |
| 8 General, with schools...... | 2,613 | 10 | — |
| 9 General, without schools | 837 | 4 | — |
| | | | 20 |
| 16 Women and women and children ............ | 926 | 4 | — |
| 4 Consumption ............. | 511 | 1 | — |
| 2 Dental ...................... | — | 1 | — |
| 3 Incurables..................... | — | 1 | — |
| 2 Cancer ...................... | 141 | 1 | — |
| 4 Paralysis and epilepsy ... | 240 | 1 | — |
| 3 Orthopœdic .................. | 113 | 1 | — |
| 2 Seamen and accidents ... | 308 | 1 | — |
| 5 Ophthalmic .................. | 197 | 1 | — |
| 5 Throat and ear ........... | 52 | 1 | — |
| 7—4 Skin and 3 Fistula, &c. | 112 | 1 | — |
| 1 Lock .......................... | 208 | 1 | — |
| 1 London fever ............. | 180 | 1 | — |
| 4 Lying-in ...................... | 132 | 1 | — |
| 7 Foreign and pay ........... | 249 | 1 | — |
| Free and part-pay dispensaries ................. | — | 1 | — |
| Provident dispensaries ... | — | 1 | — |
| | | | 20 |
| General Medical Council | — | 1 | — |
| Royal College of Physicians | — | 1 | — |
| Royal College of Surgeons | — | 1 | — |
| Society of Apothecaries... | — | 1 | — |
| General Practitioners...... | — | 1 | — |
| University for London ... | — | 1 | — |
| | | | 6 |
| London County Council... | — | 1 | 1 |
| Sunday Fund ............. | — | 1 | 1 |
| Saturday Fund ........... | — | 1 | 1 |
| | | Total ... | 49 |

### Sources of Maintenance.

It is shown by the evidence that, apart from the three endowed hospitals, the general hospitals in London are maintained principally by the legacies they receive and large donations from unexpected quarters. In most cases the subscriptions from annual subscribers do not suffice to pay the wages of the servants and nurses employed in the service of each hospital, to say nothing of the cost of maintenance and administration. It has been authoritatively stated that from £50,000 to £55,000 per annum are required to render available the 1,800 or 2,000 vacant beds which are said to exist.

One endowed hospital is maintained entirely by its endowments, but the two others are so short of funds that many beds are closed to the sick poor.

It but remains for the Committee to acknowledge the readiness with which the authorities of the medical charities and of the Poor-Law institutions have laid before them all the information desired.

June 13th, 1892.

